# Exercise alters cortico-basal ganglia network metabolic connectivity: a mesoscopic level analysis informed by anatomic parcellation defined in the mouse brain connectome

**DOI:** 10.1007/s00429-023-02659-2

**Published:** 2023-06-12

**Authors:** Zhuo Wang, Erin K. Donahue, Yumei Guo, Michael Renteln, Giselle M. Petzinger, Michael W. Jakowec, Daniel P. Holschneider

**Affiliations:** 1 Department of Psychiatry and Behavioral Sciences, University of Southern California, 1975 Zonal Avenue, KAM 400, MC9037, Los Angeles, California 90089-9037 USA; 2 Graduate Program in Neurosciences, University of Southern California, Los Angeles, California USA; 3 Present Address: Department of Neurology, University of Southern California, Los Angeles, California USA; 4 Department of Biomedical Engineering, University of Southern California, Los Angeles, California USA

**Keywords:** Mouse connectome, Functional reorganization, Caudate putamen, Motor skill learning, Brain metabolic mapping, Functional connectivity

## Abstract

The basal ganglia are important modulators of the cognitive and motor benefits of exercise. However, the neural networks underlying these benefits remain poorly understood. Our study systematically analyzed exercise-associated changes in metabolic connectivity in the cortico-basal ganglia-thalamic network during the performance of a new motor task, with regions-of-interest defined based on mesoscopic domains recently defined in the mouse brain structural connectome. Mice were trained on a motorized treadmill for six weeks or remained sedentary (control), thereafter undergoing [^14^C]-2-deoxyglucose metabolic brain mapping during wheel walking. Regional cerebral glucose uptake (rCGU) was analyzed in 3-dimensional brains reconstructed from autoradiographic brain sections using statistical parametric mapping. Metabolic connectivity was assessed by calculating inter-regional correlation of rCGU cross-sectionally across subjects within a group. Compared to controls, exercised animals showed broad decreases in rCGU in motor areas, but increases in limbic areas, as well as the visual and association cortices. In addition, exercised animals showed (i) increased positive metabolic connectivity within and between the motor cortex and caudoputamen (CP), (ii) newly emerged negative connectivity of the substantia nigra pars reticulata with the globus pallidus externus, and CP, and (iii) reduced connectivity of the prefrontal cortex (PFC). Increased metabolic connectivity in the motor circuit in the absence of increases in rCGU strongly suggests greater network efficiency, which is also supported by the reduced involvement of PFC-mediated cognitive control during the performance of a new motor task. Our study delineates exercise-associated changes in functional circuitry at the subregional level and provides a framework for understanding the effects of exercise on functions of the cortico-basal ganglia-thalamic network.

**Supplementary Information:**

The online version contains supplementary material available at 10.1007/s00429-023-02659-2.

## Introduction

It is well documented that exercise improves brain cognitive, motor, and affective functions in health and disease, and has preventive and restorative benefits in neuropsychiatric conditions, as well as in age-associated functional decline (Cotman and Berchtold 2002; Hillman et al. 2008; Petzinger et al. 2013; Gomes-Osman et al. 2018; Ludyga et al. 2020; Dauwan et al. 2021). At the molecular and cellular level, exercise effects are mediated by brain-derived neurotrophic factor and other signaling molecules, and expressed in multiple forms of brain plasticity, including neurogenesis, synaptogenesis, angiogenesis, improved mitochondrial function, altered neuroexcitability, improved or preserved white matter integrity, and enhanced neuroplasticity (Hillman et al. 2008; Nicolini et al. 2021). While these changes are believed to be beneficial to brain functions in general, how they lead to behavioral improvement remains incompletely understood. Neuroimaging investigation can offer insight of exercise effects by examining changes in functional connectivity on neural networks, thereby bridging the gap between microscopic neural substrates and behavioral outcomes (Won et al. 2021; Moore et al. 2022).

It is hypothesized that exercise, which involves motor and often cognitive tasks, recruits the cortico-basal ganglia-thalamic (CBT) network to bring about activity-dependent neuroplasticity at local and distant brain sites. Animal research has shown exercise-related metabolic, perfusion and molecular effects in individual regions of the CBT, in particular the caudoputamen, motor cortex, substantia nigra, and thalamus. Yet, only a few studies have examined functional connectivity changes in the CBT network following exercise. We previously reported that in rats with bilateral 6-hydroxydopamine lesion to the caudoputamen, exercise partially reinstated cortical sensorimotor functional connectivity lost following dopaminergic deafferentation (Peng et al. 2014) and strengthened connectivity in the CBT and cerebellar-thalamocortical circuits (Wang et al. 2015). Ji et al. (2017) reported an exercise-associated increase in resting-state functional connectivity between putamen and thalamus in human subjects. Increased resting-state functional connectivity of the motor cortex has been reported in normal volunteers following several minutes (McNamara et al. 2007; Sun et al. 2007) or 4 weeks of motor training (Ma et al. 2010). Exercise-associated reduction in resting-state functional connectivity of the basal ganglia has also been reported (Magon et al. 2016; Tao et al. 2017). These studies typically report functional connectivity changes of large areas such as the whole putamen, masking subregional (mesoscopic level) heterogeneity in network structure and function. To our best knowledge, there has not been a systematic analysis of exercise-associated changes in functional connectivity at the mesoscopic level over the CBT network.

Recent development in the mouse brain connectome has brought unprecedented, detailed information on the structural organization of the CBT network. In particular, newly identified domains at the mesoscopic level have been defined for key structures of the basal ganglia based on patterns of axonal projections. Dong and coworkers have subdivided the caudoputamen, the globus pallidus externus and the substantia nigra pars reticulata into multiple domains based on the structural cortico-striatal projectome (Hintiryan et al. 2016), and projectome within the basal ganglia (Foster et al. 2021). This connectomic information creates a framework for systematic functional connectivity analysis of the basal ganglia. The current study applied the classic [^14^C]-2-deoxyglucose (2DG) uptake autoradiographic method of cerebral metabolic mapping to examine functional reorganization in the CBT network in response to chronic exercise. The well-established 2DG method is particularly suitable for high-resolution mapping in awake, freely-moving animals. Following 6 weeks of exercise training on a treadmill, glucose uptake was mapped in animals performing a novel, wheel-walking task. There is evidence that chronic exercise promotes motor learning capacity (Li and Spitzer 2020), with associated changes in functional brain activation correlating with changes in aerobic fitness (Duchesne et al. 2016). Prior work has not examined correlation between aerobic fitness and functional connectivity in the CBT network, which is implicated in new motor learning (Dayan and Cohen 2011). We applied the domain definitions of Dong and coworkers in a region-of-interest approach to investigate exercise-associated changes in functional connectivity of the network. Specifically, inter-regional correlations of regional cerebral glucose uptake were calculated cross-sectionally across animals within a group to assess metabolic connectivity, which was interpreted as a measure of functional connectivity. Our findings provide new insight into exercise-associated alterations in functional interactions within individual structures and across the CBT network. Functional neuroimaging research that harnesses state-of-the-art anatomic connectomic information can bridge the current knowledge gap in understanding exercise effects between the microscopic and behavioral levels.

## Materials and methods

### Animals

Male C57BL/6 J mice were purchased from Jackson Laboratory (Bar Harbor, Maine, USA) and housed in groups of 4–5 per cage on direct woodchip bedding at the University of Southern California vivarium. Animals had ad libitum access to laboratory rodent chow and water and were maintained on a 12 h light/12 h dark cycle (lights on at 0700 and off at 1900 h). All experimental procedures involving animals were approved by the Institutional Animal Care and Use Committee at the University of Southern California (Protocol # 21,044) and carried out in compliance with the National Institutes of Health Guide for the Care and Use of Laboratory Animals, 8th Edition, 2011.

### ***Overview (***Fig. 1***)***

**Fig. 1:**
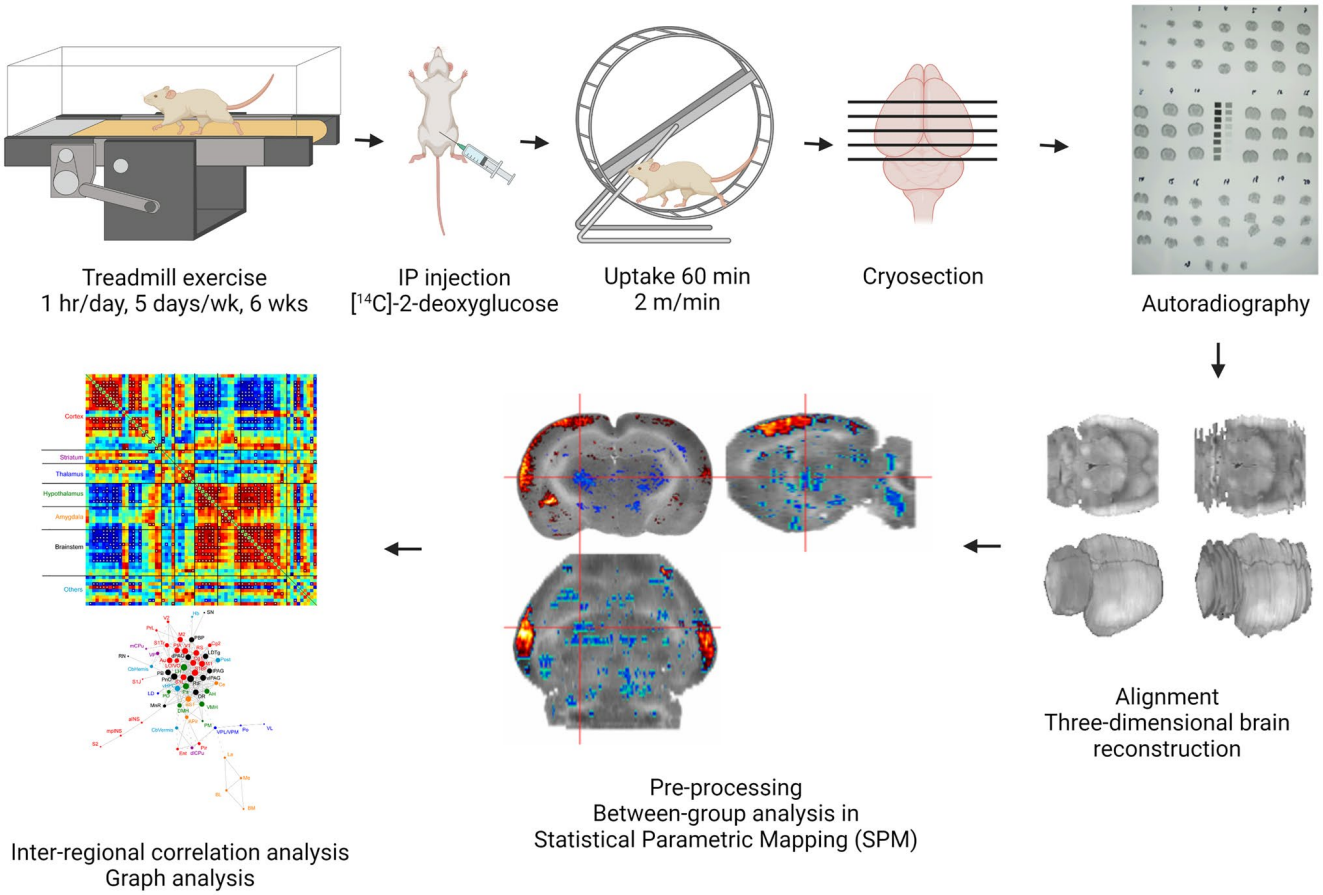
Experiment protocol. Created with BioRender.com

A total of 20 mice were randomized into two groups (*n* = 10/group, aged 4—6 months by the end of experiment): control (sedentary) and exercise. Animals received 6 weeks of exercise training on a motorized treadmill or sedentary treatment, followed by [^14^C]-2DG cerebral metabolic mapping while the animals walked in a wheel, a new motor task. The brains were cryosectioned into coronal slices, which were subsequently exposed to films for autoradiography. Digitized images of brain slices were used to reconstruct three-dimensional (3D) brains. These 3D brains were preprocessed using the Statistical Parametric Mapping (SPM) software, followed by statistical tests for between-group differences in regional cerebral glucose uptake (rCGU). For metabolic connectivity analysis, regions of interests (ROIs) were created to represent mesoscopic-level domains of the basal ganglia structures as defined by the mouse structural connectome (Hintiryan et al. 2016) (Foster et al. 2021) and select cortical and thalamic structures associated with motor and cognitive functions as represented in the mouse brain atlases (Dong 2008; Franklin and Paxinos 2008). Pairwise inter-regional correlation matrices were calculated cross-sectionally across animals within each group to assess metabolic connectivity. Network organization was further analyzed using graph theory analytic tools.

### Treadmill exercise and control treatment

Mice were exercised on motorized, horizontal treadmills (EXER-6, Columbus Instruments, Columbus, OH, USA) for 1 h/day, 5 days/week over 6 weeks, as previously described (Lundquist et al. 2019) with slight modifications. The exercise protocol included a 20-min warm-up phase when a base speed of 5 m/min was ramped up incrementally every 5 min to a top speed, a 10-min running at the top speed, a 5 min walk break at 5 m/min, another 10-min running at the top speed, and a final 20-min cool-down phase when speed was ramped down every 5 min to a final speed of 5 m/min. During the first several days of exercise, top speed was incrementally increased to allow the mice to adapt to running at higher speed. The final top speed of 19 m/min was introduced on day 8 of exercise. Control (sedentary) mice were kept in home cages placed on a plastic barrier overlying the motorized treadmills for the same duration (1 h/day, 5 days/week, 6 weeks) so that they were subjected to similar vibrations and auditory stimulation.

### Autoradiographic glucose metabolic mapping during wheel walking

The autoradiographic 2DG uptake method is a well-established approach to functional brain mapping based on a tight coupling between neural activity and metabolism. It is particularly suitable in awake, freely-moving animals, and hence can be applied to exploration of network connectivity in the behaving animal. The protocol is as previously described with modifications (Sokoloff et al. 1977; Holschneider et al. 2019; Needham et al. 2022). For 2 days prior to the day of 2DG mapping, mice were individually familiarized to walk in a closed wheel for 10 min/day at a modest speed of 2 m/min (3.3 cm/s) on a motorized wheel bed (Model 80805A, Lafayette Instrument, Lafayette, IN, USA)–a new motor task that can be learned by all animals at this low speed. Using the wheel-walking task avoided the confound of different familiarity to the treadmill between the exercise and control group. The walking wheel (Model 80,801) had an internal diameter of 15 cm and width of 5.7 cm, and was equipped with a safety mesh netting (Model 80801MSH25) to prevent the animal’s tail from being pinched. Animals were brought to the experimental suite 16 h before mapping experiments and were fasted of food overnight with water ad libitum.

For 2DG uptake, the animal was administered IP [^14^C]-2DG (cat # MC355, Moravek Inc., Brea, CA, USA) at 0.3 µCi/g bodyweight in 0.53 ml normal saline. The animal was subsequently placed inside the closed walking wheel to walk at 2 m/min for 60 min to allow uptake of the tracer. At the end of walking, the animal was euthanized by cervical dislocation and the brain was extracted and flash-frozen in methylbutane over dry ice (about – 55 °C). The brains were later serially sectioned into 20-μm coronal slices, sampled with a 140-μm inter-slice distance, in a cryostat at – 18 °C (Mikron HM550 OMP, Thermo Fisher Scientific, Waltham, MA, USA). Slices were heat-dried on glass slides and exposed to Kodak Biomax MR diagnostic film (Eastman Kodak, Rochester, NY, USA) for 3 days at room temperature. Autoradiographs were then digitized on an 8-bit grey scale using a voltage-stabilized light box (Northern Light R95 Precision Illuminator, Imaging Research Inc., St. Catharines, Ontario, Canada) and a Retiga 4000R charge-coupled device monochrome camera (QImaging of Teledyne Photometrics, Tucson, AZ, USA). Optical density in the digitized autoradiographs was used as a measure of cumulative glucose uptake in the brain.

### Whole-brain analysis of regional cerebral glucose uptake

We and others have developed a pipeline to create 3D rodent brains using autoradiographs of serial brain sections, which were then preprocessed and analyzed using the Positron Emission Tomography (PET) module in the SPM package (Wellcome Centre for Neuroimaging, University College London, London, UK) for cross-sectional analysis of rodent autoradiographic cerebral blood flow and cerebral glucose uptake data (Nguyen et al. 2004; Dubois et al. 2010; Needham et al. 2022). For each animal, a 3D brain was reconstructed from 66 digitized, autoradiographic images of coronal sections (voxel size: 40 μm × 140 μm × 40 μm) using our prior methods (Nguyen et al. 2004). Sections were selected starting at + 2.4 mm anterior to the internal landmark of bregma. Adjacent sections were aligned using TurboReg, an automated pixel-based registration algorithm implemented in ImageJ (v.1.35, https://imagej.nih.gov/ij/index.html). This algorithm registered each section sequentially to the previous section using a non-warping geometric model that included rotations, rigid-body transformation and nearest-neighbor interpolation. For preprocessing, one mouse brain was selected as reference. All brains were spatially normalized to the reference brain in SPM (version 5). Spatial normalization consisted of applying a 12-parameter affine transformation followed by a nonlinear spatial normalization using 3D discrete cosine transforms. All normalized brains were then averaged to create a final brain template. Each original brain was then spatially normalized to the template. Final normalized brains were smoothed with a Gaussian kernel (full-width at half-maximum = 240 μm × 420 μm × 240 μm) to improve the signal-to-noise ratio. Proportional scaling was used to scale voxel-wise optical density so that the whole-brain average was the same across animals. Global cerebral glucose uptake is believed to change very little with normal physiological alterations in cerebral functional activity (Sokoloff 1991). This would be expected during the slow walking task in this study.

Unbiased, voxel-by-voxel Student’s *t-*tests between the exercise and control group were performed across the whole brain to access changes in rCGU following exercise using SPM. Threshold for statistical significance was set at *P* < 0.05 at the voxel level with an extent threshold of 200 contiguous significant voxels. This combination reflected a balanced approach to control both Type I and Type II errors. The minimum cluster criterion was applied to avoid basing our results on significance at a single or a small number of suprathreshold voxels. Brain regions were identified according to mouse brain atlases (Dong 2008; Franklin and Paxinos 2008). Color-coded functional overlays showing statistically significant changes in rCGU were displayed over coronal sections of the template brain in MRIcro (v.1.40, https://people.cas.sc.edu/rorden/mricro/mricro.html).

### Metabolic connectivity analysis of the cortico-basal ganglia-thalamic-cortical network

**Fig. 2:**
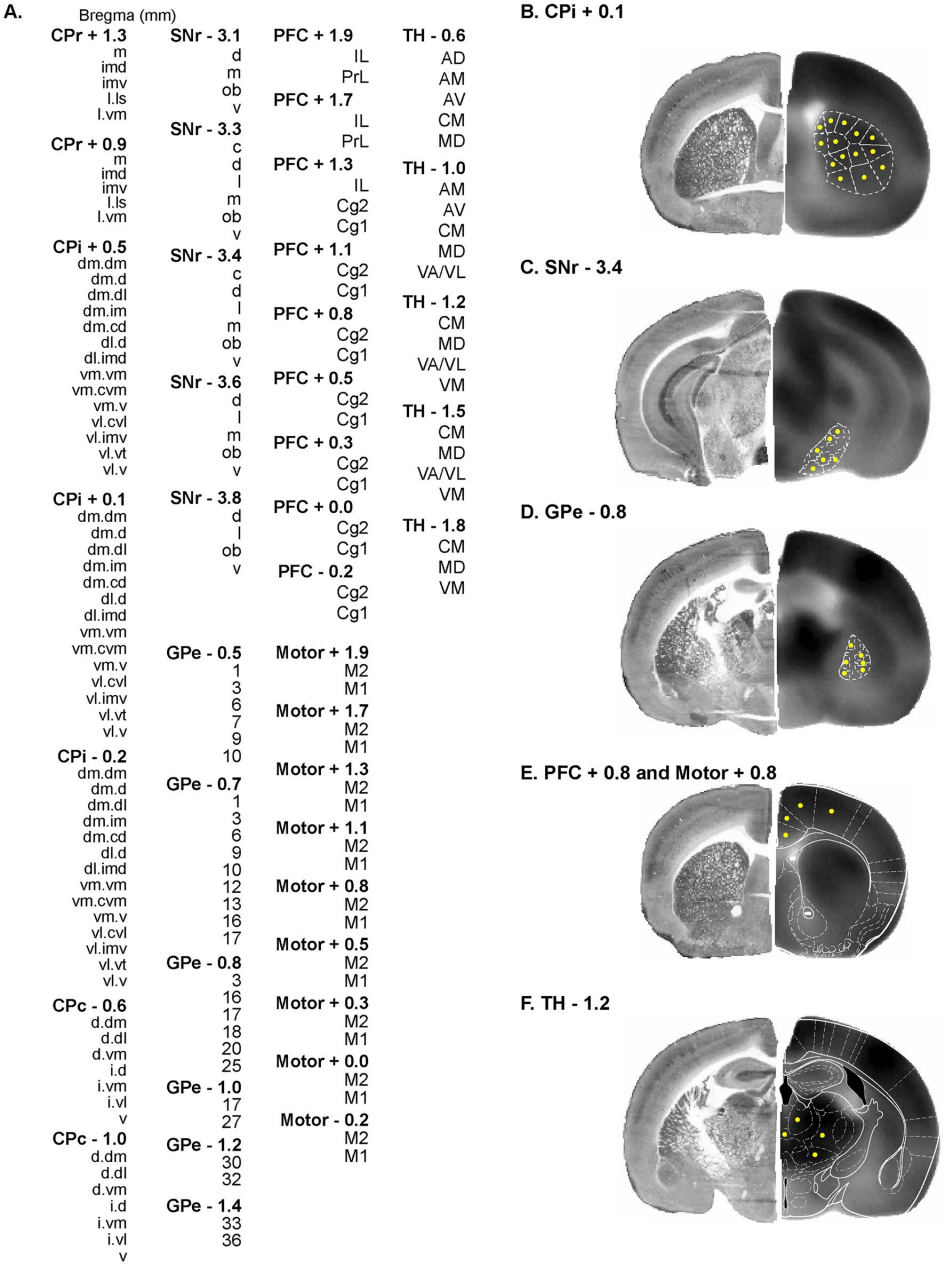
Definition of regions of interest (ROIs) for analysis of metabolic connectivity. **A** List of 176 ROIs defined. A group of ROIs are defined for each structure at a given bregma level, e.g. CPr + 1.3, rostral caudoputamen at bregma + 1.3 mm. CPi/CPc, intermediate/caudal caudoputamen; SNr, substantia nigra pars reticulata; GPe, globus pallidus externus; PFC, prefrontal cortex (Cg1/Cg2, cingulate area 1/2. IL, infralimbic. PrL, prelimbic); M1/M2, primary/secondary motor cortex; TH, thalamic nuclei (AD, anterodorsal. AM, anteromedial. AV, anteroventral. CM, central medial. MD, mediodorsal. VA/VL, ventral anterior/ventrolateral. VM, ventromedial). **B** ROI definition for CPi domains at bregma + 0.1 mm as defined in (Hintiryan et al. 2016)(see also Fig. 8). The hemisphere on the left shows histochemical staining for cytochrome oxidase from a representative brain used to assist brain area identification. The hemisphere on the right is a coronal section of the template brain showing [^14^C]-2-deoxyglucose uptake. ROIs (yellow circles) are drawn near the approximate center of each domain. **C, D** ROI definition for SNr domains at bregma – 3.4 mm and GPe domains At – 0.8 mm according to Foster et al. (2021)(see also Fig. 9). Some domains too small in size were not included in this analysis. **E** ROI definition for PFC and motor cortex at bregma + 0.8 mm. White outlines are modified from the mouse brain atlas (Franklin and Paxinos 2008). **F** ROI definition for TH at bregma – 1.2 mm

We took an ROI approach to assessing brain metabolic connectivity. A total of 176 ROIs were defined for six structures critical to motor and cognitive functions, including the caudoputamen (CP), globus pallidus externus (GPe), substantia nigra pars reticulata (SNr), prefrontal cortex (PFC, including infralimbic, IL; prelimbic, PrL; cingulate area 1 and 2, Cg1 and Cg2), motor cortex (including primary and secondary motor, M1 and M2), and thalamic nuclei (anterodorsal, AD; anteromedial, AM; anteroventral, AV; central medial, CM; mediodorsal, MD; ventral anterior/ventrolateral, VA/VL; ventromedial, VM) (Fig. 2). ROIs were drawn on coronal sections of the template brain in MRIcro. A group of ROIs were defined for each structure at a given bregma level, e.g. 5 ROIs were defined for the rostral caudoputamen at bregma + 1.3 mm (CPr + 1.3, Fig. 2A). ROIs for CP, SNr, and GPe were based on mesoscopic-level domain definitions as set forth in the mouse brain anatomic connectome (Hintiryan et al. 2016; Foster et al. 2021). Domain definition maps were transcribed to a visual template. Overlay of this template on to coronal sections of the template brain allowed ROI definition in a standardized manner. Circular ROIs were drawn near the approximate center of each domain. Some domains too small in size in the SNr and GPe were not included in this analysis. ROIs for PFC, motor cortex, and thalamus were based on the mouse brain atlas (Dong 2008; Franklin and Paxinos 2008). A second set of brain slices collected adjacent to each autoradiographic section were histochemically stained for cytochrome oxidase. These histochemical images showing cytoarchitectural details were used to assist in the brain area identification in the autoradiographic images.

Mean optical density of all voxels in each ROI was calculated from each mouse brain using the MarsBaR toolbox for SPM (v.0.42, http://marsbar.sourceforge.net). We applied pairwise inter-regional correlation analysis to investigate brain metabolic connectivity. This is a well-established method, which has been applied to analyze rodent brain mapping data of multiple modalities, including autoradiographic 2DG (Soncrant et al. 1986; Nair and Gonzalez-Lima 1999), autoradiographic cerebral blood flow (Wang et al. 2011), cytochrome oxidase histochemistry (Shumake et al. 2004; Fidalgo et al. 2011), activity regulated c-*fos* gene expression (Wheeler et al. 2013), and functional magnetic resonance imaging (fMRI) data (Schwarz et al. 2007). In this approach, correlations were calculated cross-sectionally (at a single time point) across subjects within a group. Similar metabolic connectivity analyses are often performed in PET studies. The method precluded analysis of temporal dynamics of functional brain activation and differed from functional connectivity based on within subject cross correlation analysis used in fMRI studies. While these different brain mapping modalities and analytic methods provide complementary information on brain functional connectivity in general, in comparing the results one should consider possible influence of the differences in the time scales of data sampling (Di and Biswal 2012; Buckner et al. 2013; Hutchison et al. 2013; Wehrl et al. 2013).

Pearson's correlation coefficients between pairs of ROIs were calculated across subjects within a group in Matlab (Mathworks, Inc., Natick, MA, USA) to construct a correlation matrix. The matrices were visualized as heatmaps after Fisher's r-to-Z-transformation of the correlation coefficients. Statistical significance of between-group difference in correlation coefficients was evaluated using the Fisher’s Z-test (Fisher 1992):

$$Z = \frac{{\frac{1}{2}\ln \frac{{1 + r_{1} }}{{1 - r_{1} }} - \frac{1}{2}\ln \frac{{1 + r_{2} }}{{1 - r_{2} }}}}{{\sqrt {\frac{1}{{n_{1} - 3}} + \frac{1}{{n_{2} - 3}}} }}$$

where *r*_1_ and *r*_2_ denote correlation coefficient in the exercise and control group, respectively, while *n*_1_ and *n*_2_ denote sample size for these groups. A positive *Z* value indicates that *r*_1_ is greater than *r*_2_.

To control Type I error caused by the large number of correlations computed, we implemented a jackknife procedure following (Barrett et al. 2003). For a group of *n* subjects, *n* iterations were performed in which one subject was dropped sequentially and the correlation matrix recalculated with the remaining *n*–1 subjects. A correlation was considered ‘reliably’ significant only if it was statistically significant (*P* < 0.05) in all iterations. For the Fisher's z-test, 2*n* iterations were performed in which one subject was dropped sequentially from either group and the *Z* matrix recalculated between the groups, with *n* and *n*–1 subjects. Difference in correlation coefficients was considered significant only if it was statistically significant (*P* < 0.05) in all 2*n* iterations.

To further facilitate between-group comparison of within and between structure metabolic connectivity, we defined *connectivity density* as the number of connections (statistically significant correlations after jackknife correction) expressed as a percentage of the total number of ROI pairs. For example, the caudoputamen has 66 ROIs and thus a total of 2,145 (66*65/2) ROI pairs within the structure. There were 151 statistically significant, positive correlations within the caudoputamen in the exercise group. Therefore, connectivity density was + 7.04% (151/2145×100%). For another example, the total number of ROI pairs between the caudoputamen (66 ROIs) and the thalamus (21 ROIs) is 1,386 (66×21). There were 10 significant, positive correlations between these structures. Connectivity density was + 0.72% (10/1386×100%). Connectivity density was calculated separately for positive and negative correlations.

To delineate organization of the functional networks identified by statistically significant correlations (jackknife corrected) in the correlation matrices, graph theoretical analysis was performed as previously described (Wang et al. 2012) with the Pajek software (version 2.03, http://mrvar.fdv.uni-lj.si/pajek/) (Mrvar and Batagelj 2016). Each ROI was represented by a node in a graph, and two nodes with statistically significant correlation (positive or negative) were linked by an edge. A Kamada–Kawai algorithm was implemented to arrange (energize) the graph such that strongly connected nodes were placed closer to each other, while weakly connected nodes were placed further apart. Such energized graph provided an intuitive visualization of the network organization. To identify network hubs, connectivity degree of each node was calculated as the number of edges linking it to the rest of the network. Intuitively, nodes with higher degrees were more central in the network organization. Nodes with degrees ranked in the top 10% were considered hubs.

### Cytochrome oxidase histochemical staining

A second set of brain slices adjacent to sections of the autoradiographic reference brain were collected and histochemically stained for cytochrome oxidase. Histochemical images showing cytoarchitectural details were used to assist brain area identification in the autoradiographic images. Histochemical staining was undertaken with a protocol adapted from (Puga et al. 2007). In brief, staining proceeded at 4 °C as follows: (a) Pre-incubation fixation for 5 min in a phosphate buffer (0.1 M, pH 7.6) containing 10% sucrose and 0.5% glutaraldehyde; (b) Rinse 5 min × 3 times with phosphate buffer (0.1 M, pH 7.6) containing 10% sucrose; (c) Color intensification for 10 min in a Tris buffer (0.05 M, pH 7.6) containing 275 mg/L cobalt chloride (CoCl_2_), 0.5% DMSO, and 10% sucrose; (d) Rinse for 5 min with phosphate buffer (0.1 M, pH 7.6) containing 10% sucrose; (e) Staining incubation for 60 min at 37 °C with O_2_ bubbling in 700 ml 0.1 M phosphate buffer containing 10% sucrose, 14 mg of catalase, 350 mg of diaminobenzidine tetrahydrochloride (DAB), 52.5 mg cytochrome c, and 1.75 ml of DMSO; (f) Stain termination/fixation at RT for 30 min in 0.1 M phosphate buffer containing 10% sucrose and 10% formalin; (g) Dehydration with ethanol and clearing with xylene. Slides were coverslipped with Permount. Histological images were digitized and used to reconstruct a 3D brain as described above for autoradiographic images.

## Results

### Effects of exercise on regional cerebral glucose uptake

**Fig. 3:**
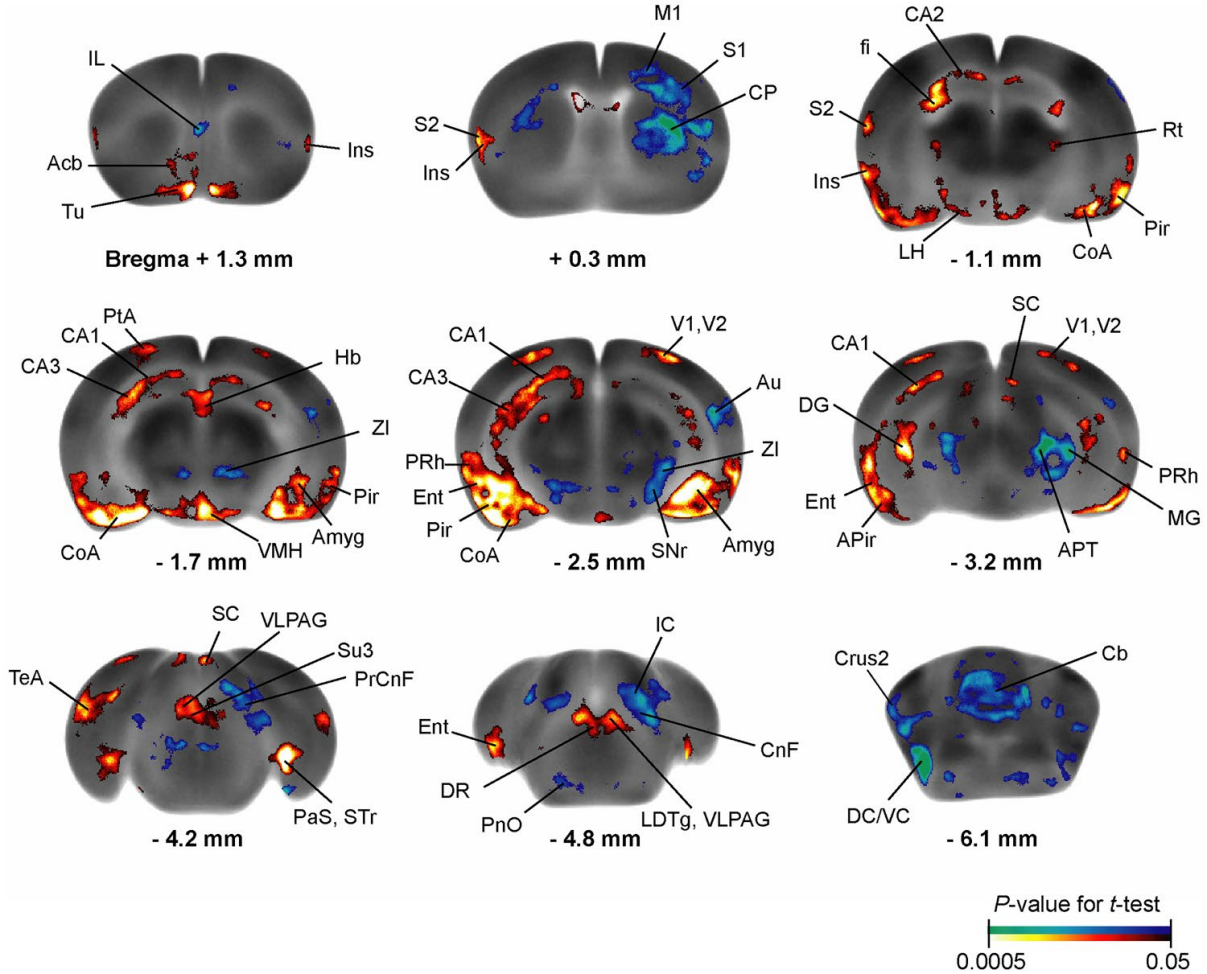
Exercise effects on regional cerebral glucose uptake (rCGU). Color-coded overlays show statistically significant increases (red) and decreases (blue) in rCGU in the exercise compared to the control group (*n* = 10/group, *P* < 0.05 and extent threshold > 200 contiguous voxels, Student's *t*-test). Shown are representative coronal sections of the template brain. Regions are identified according to the mouse brain atlas (Dong 2008; Franklin and Paxinos 2008). *Acb* nucleus (n) accumbens, *Amyg* amygdala, *APir* amygdalopiriform transition, *APT* anterior pretectal n., *Au* auditory cortex (cx), *CA1/CA2/CA3* field CA1/CA2/CA3 hippocampus, *CnF* cuneiform n., *CoA* cortical amygdala, *CP* caudoputamen, *Crus2*, crus 2 of the ansiform lobule; *DC/VC* dorsal/ventral cochlear n., *DG* dentate gyrus, *DR* dorsal raphe n., *Ent* entorhinal cx, *fi* fimbria, *Hb* habenular n., *IC* inferior colliculus, *IL* infralimbic cx, *Ins* insular cx, *LDTg* laterodorsal tegmental n., *LH* lateral hypothalamus, *M1* primary motor cx, *MG* medial geniculate n., *PaS* parasubiculum, *Pir* piriform cx, *PnO* pontine reticular n. oral part, *PrCnF* precuneiform area, *PRh* perirhinal cx, *PtA* parietal association cx, *Rt* reticular thalamic n.; *S1/S2* primary/secondary somatosensory cx, *SC* superior colliculus, *SNr* substantia nigra pars reticulate, *STr* subiculum transition area, *Su3* supraoculomotor periaqueductal gray, *TeA* temporal association cx, *Tu* olfactory tubercle; *V1/V2* primary/secondary visual cx, *VLPAG* ventrolateral periaqueductal gray, *VMH* ventromedial hypothalamic n., *ZI* zona incerta

Exercise resulted in broad changes across the CBT network during wheel walking (Fig. 3). Significant rCGU decreases were seen in the exercise compared to control group in motor regions, including primary motor cortex, the basal ganglia (intermediate CP, SNr), zona incerta, cerebellum (vermis, crus 2 of the ansiform lobule), as well as associated motor regions (cuneiform nucleus, precuneiform area), and sensory regions, including cortical areas (auditory, infralimbic, primary somatosensory), medial geniculate nucleus, inferior colliculus, dorsal and ventral cochlear nucleus, anterior pretectal nucleus, and pontine reticular nucleus oral part. Exercised compared to control animals showed statistically significant rCGU increases in the limbic areas, including the hippocampus (CA1, CA2, CA3 fields, dentate gyrus, fimbria), parasubiculum, entorhinal cortex, piriform cortex, insula, amygdala, hypothalamus (lateral and ventromedial), nucleus accumbens, dorsal raphe, periaqueductal gray (ventrolateral, supraoculomotor) (*P* < 0.05, ≥ 200 significant contiguous voxels). Significant increases were also seen in the secondary somatosensory, primary and secondary visual, perirhinal, parietal association, and temporal association cortices, as well as in the olfactory tubercle, reticular thalamic nucleus, habenular nucleus, superior colliculus, and laterodorsal tegmental nucleus.

Variability in the rCGU data between individual animals in selected ROIs can be viewed in Supplementary Fig. S1.

### Sedentary control group: Metabolic connectivity of the cortico-basal ganglia-thalamic network

**Fig. 4:**
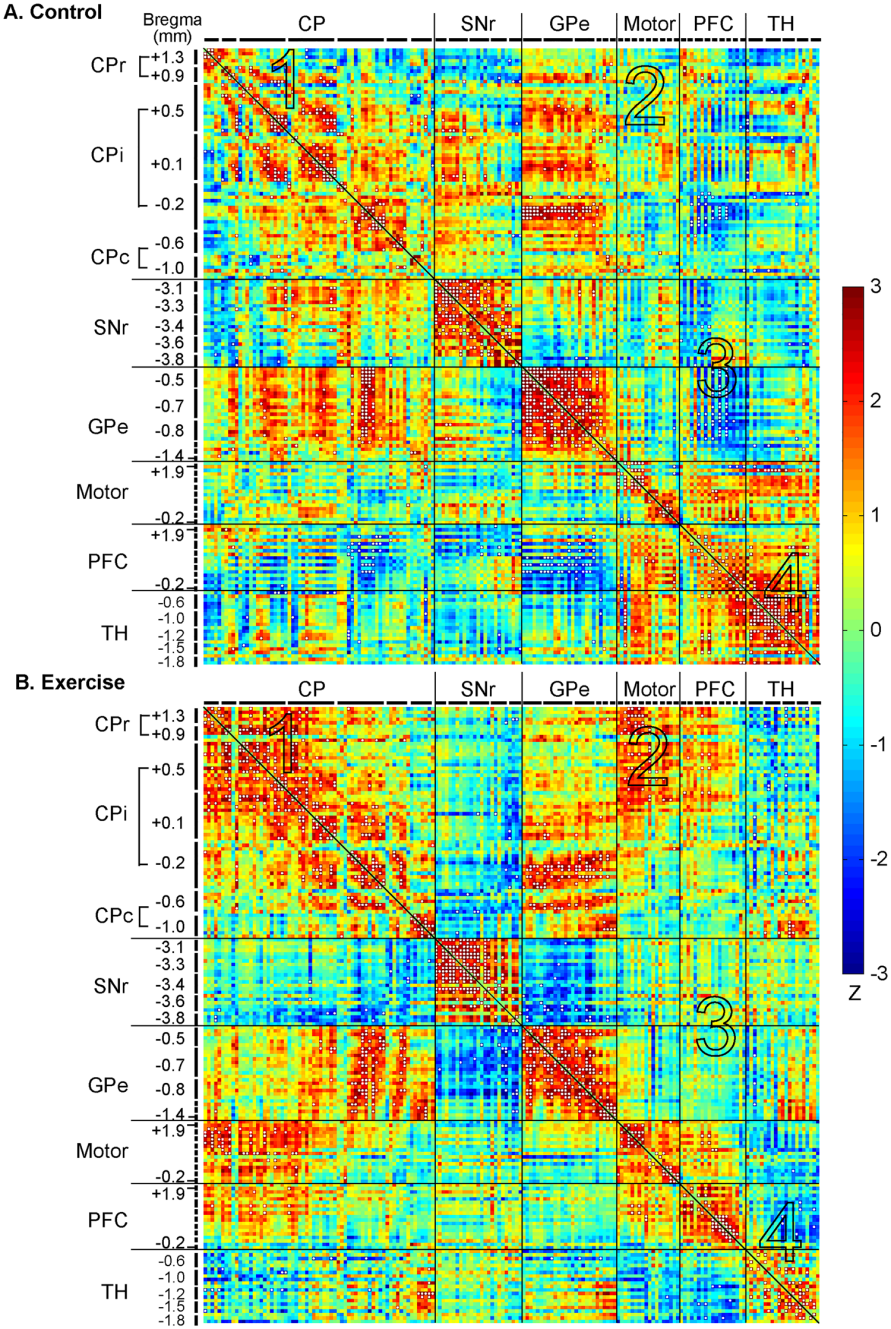
Exercise effects on metabolic connectivity of the cortico-basal ganglia-thalamic-cortical network. **A** Inter-regional correlation matrix shows metabolic connectivity patterns in the non-exercised control group (*n* = 10). Pearson's correlation coefficients following Fisher's r-to-Z transformation are color-coded with positive and negative values shown in warm and cold colors, respectively. The matrix is symmetric across the diagonal line from upper left to lower right. Significant correlations (*P* < 0.05) following the jackknife procedure are marked with white dots and interpreted as functional connections. Regions of interest are arranged in the sequence shown in Fig. 2A. They are grouped by structure, and further grouped by bregma level (marked by short, black lines) and arranged from rostral to caudal within each structure. **B** Inter-regional correlation matrix in the exercise group (*n* = 10). *CP* caudoputamen (CPr/CPi/CPc, rostral/intermediate/caudal), *SNr* substantia nigra pars reticulate, *GPe* globus pallidus externus, *PFC* prefrontal cortex, *TH* thalamus. The large numbers embedded in the heatmaps label pathways showing major exercise effects: 1, within the CP; 2, between the CP and motor cortex; 3, between the PFC and basal ganglia; 4, within the thalamus, between the thalamus and motor cortex, between the thalamus and PFC

In the control group (Fig. 4A and Table 1), metabolic connectivity of the network was characterized by strong, positive within structure connectivity in the SNr (+ 21.00%, positive connectivity density), GPe (+ 32.48%), and thalamus (+ 26.67%); and modest, primarily positive within structure connectivity in the CP (+ 4.38%), motor cortex (+ 13.07%), and PFC (+ 8.77%) (Fig. 4A, along the diagonal line). Primarily positive between structure connectivity was seen between the CP and GPe (+ 4.21%), motor cortex and PFC (+ 1.75%), motor cortex and thalamus (+ 2.38%), and PFC and thalamus (+ 4.26%). The PFC showed primarily negative connectivity with the basal ganglia: with CP (− 2.15%, negative connectivity density), with SNr (− 1.26%), and with GPe (− 8.77%) (Table 2).

**Table 1:** Within structure metabolic connectivity density. Connectivity density is defined as the number of connections (statistically significant correlations after jackknife correction) expressed as a percentage of the total number of ROI pairs in the structure. *n* = 10/group

		Caudo-putamen	Substantia nigra pars reticulata	Globus pallidus externus	Motor cortex	Prefrontal cortex	Thalamus
Positive (%)	Exercise	+ 7.04	+ 28.33	+ 24.79	+ 12.42	+ 11.70	+ 11.90
	Control	+ 4.38	+ 21.00	+ 32.48	+ 13.07	+ 8.77	+ 26.67
Negative (%)	Exercise						
	Control	− 0.51			− 1.31

**Table 2:**
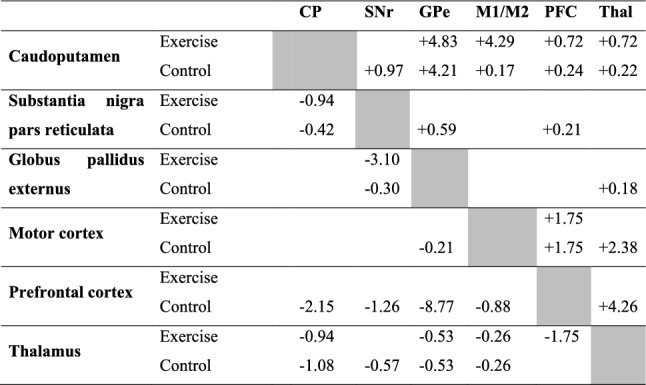
Between structure metabolic connectivity density. The top-right half of the table shows positive connectivity densities, which are marked with "+". The lower-left half of the table shows negative connectivity densities, marked with "-". Connectivity density is defined as the number of connections (statistically significant correlations after jackknife correction) expressed as a percentage of the total number of ROI pairs between two specified brain structures. n = 10/group

The top-right half of the table shows positive connectivity densities, which are marked with " + ". The lower-left half of the table shows negative connectivity densities, marked with "−". Connectivity density is defined as the number of connections (statistically significant correlations after jackknife correction) expressed as a percentage of the total number of ROI pairs between two specified brain structures. *n* = 10/group.

**Fig. 5:**
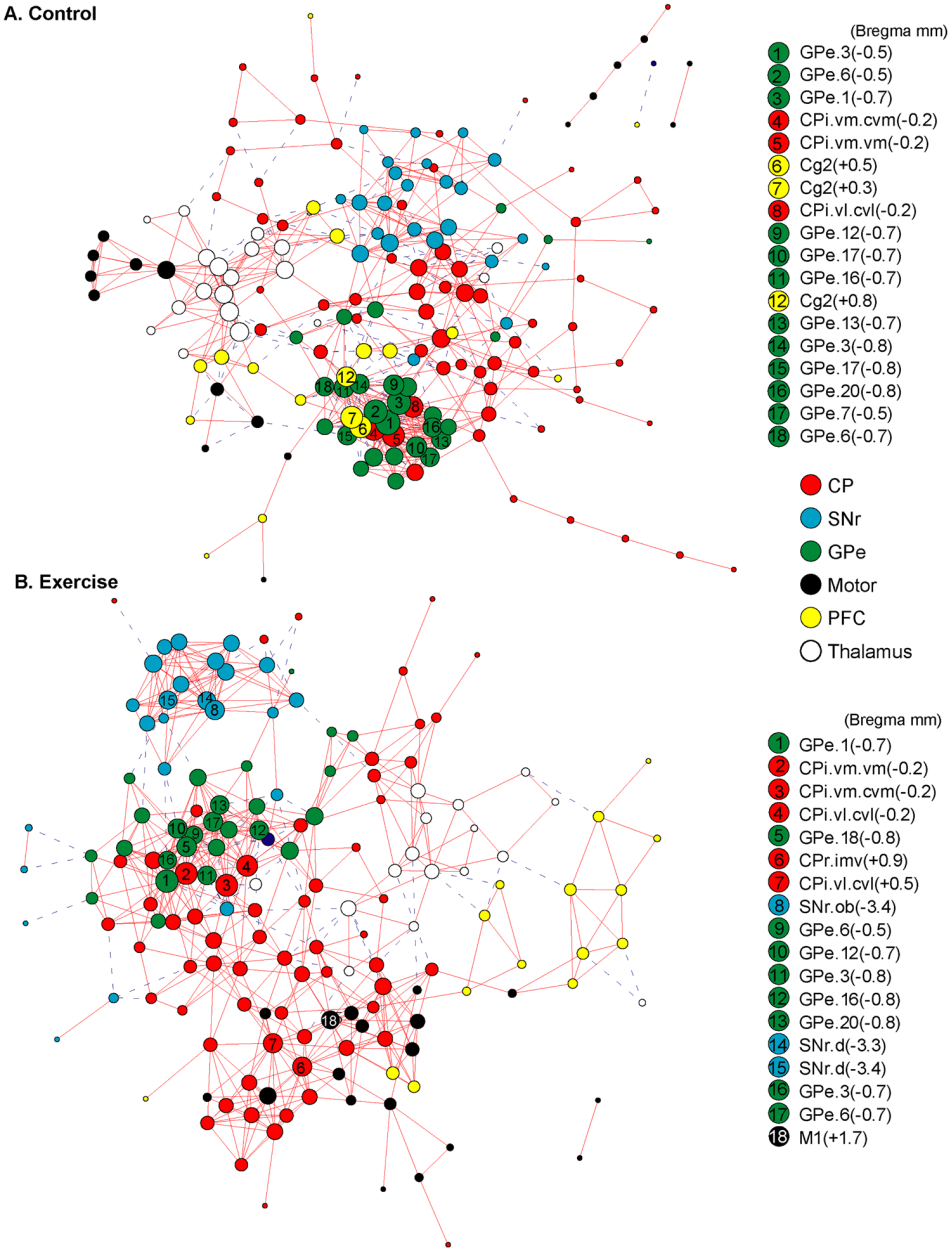
Exercise effects on the organization of connectivity graphs of the cortico-basal ganglia-thalamic-cortical network. **A** The metabolic connectivity network of the non-exercise control group (*n* = 10) is represented with a graph, in which nodes represent regions of interest (ROIs) and edges represent significant correlations after the jackknife correction. Solid red lines denote significant positive correlations, whereas dashed blue lines denote significant negative correlations. The graph is energized using the Kamada–Kawai algorithm that places strongly connected nodes closer to each other while keeping weakly connected nodes further apart. The size of each node (in area) is proportional to its degree, a measurement of the number of connections linking the node to other nodes in the network. ROIs with the highest degree (top 10%) are considered hubs of the network and labeled with their ranking numbers. Nodes are color-coded to facilitate identification of nodes belonging to the same structure. **B** Connectivity graph of the exercise group (*n* = 10). *CP* caudoputamen, *SNr* substantia nigra pars reticulate, *GPe* globus pallidus externus, *PFC* prefrontal cortex. For locations of ROIs see also Figs. 2, 8, 9

Figure 5A shows a connectivity graph of the control group based on the correlation matrix. The graph was energized using the Kamada-Kawai algorithm to help visualize network organization. Consistent with their high within structure connectivity, the SNr (blue nodes), GPe (green), and thalamus (white) each formed a cluster. The motor cortex nodes (black) were closely connected with the thalamic cluster. The CP nodes (red) and PFC nodes (yellow) were both more scattered, making connections with all other clusters, while showing a particularly high level of integration with the GPe cluster. Nodes with the highest connectivity degrees (top 10%) were considered network hubs and included 12 GPe nodes, 3 intermediate CP (CPi) nodes, and 3 PFC nodes (Cg2).

### Exercise group: Metabolic connectivity of the cortico-basal ganglia-thalamic network

**Fig. 6:**
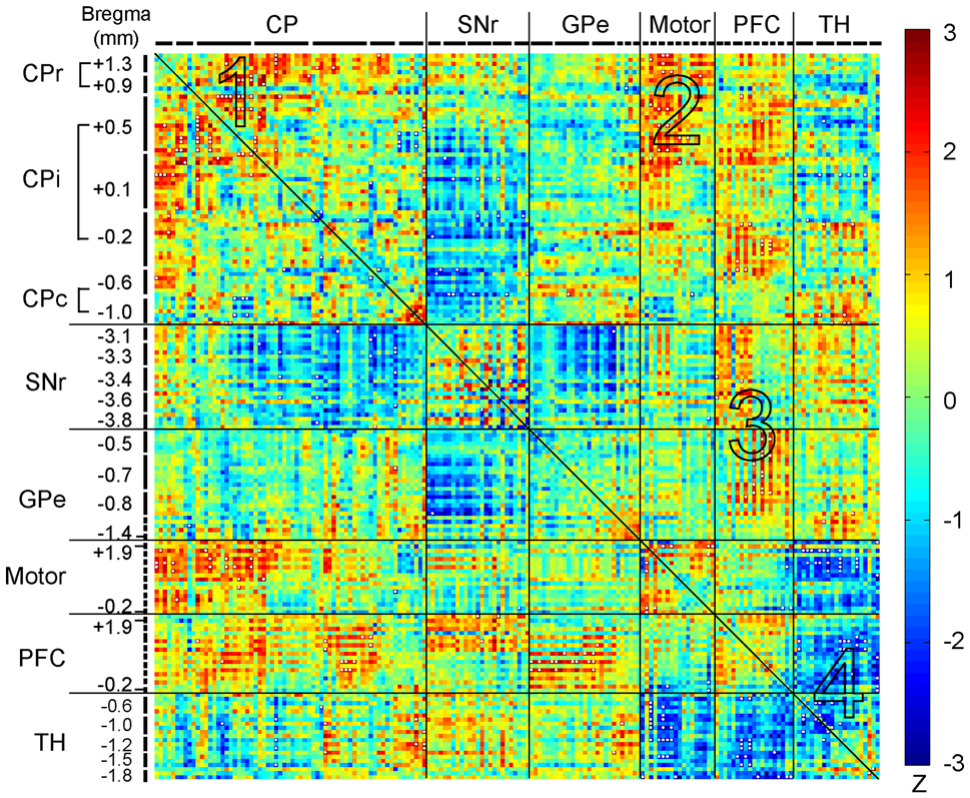
Exercise vs. control group: Changes in metabolic connectivity. The matrix of Fisher's Z-test for differences in Pearson's correlation coefficients (*r*) between exercise and control groups (*n* = 10/group). Positive/negative *Z* values indicate greater/smaller *r* in the exercise compared to control group. Significant between-group differences (*P* < 0.05) following the jackknife procedure were marked with white dots. *CP* caudoputamen (CPr/CPi/CPc, rostral/intermediate/caudal), *SNr* substantia nigra pars reticulate, *GPe* globus pallidus externus, *PFC* prefrontal cortex, *TH* thalamus. The large numbers embedded in the heatmap label pathways showing major exercise effects: 1, within the CP, 2 between the CP and motor cortex, 3 between the PFC and basal ganglia; 4 within the thalamus, between the thalamus and motor cortex, between the thalamus and PFC

The exercise compared to control group showed broad changes in metabolic connectivity (Fig. 4B, Fig. 6, and Tables 1, 2) and network organization (Fig. 5B). The following major differences comparing the exercise and control group correspond to areas numbered 1 through 4 in Fig. 4 and Fig. 6. (1) Connectivity density within the caudoputamen increased from + 4.38% in the control to + 7.04% in the exercise group. Increased connections were mainly located in rostral (CPr) and dorsal aspect of intermediate caudoputamen (CPi). (2) In the exercise group, new, positive connections were formed between CPr/CPi and motor cortex. (3) Negative connectivity between PFC and the basal ganglia observed predominantly in the control group was largely absent in the exercise group. (4) Connectivity density within the thalamus decreased from + 26.67% in the control to + 11.90% in the exercise group. Positive thalamus-motor cortex connectivity (+ 2.38%) and thalamus-PFC connectivity (+ 4.26%) seen in the control group were absent in the exercise group.

Figure 5B shows energized connectivity graph of the exercise group. The PFC nodes (yellow) were marginalized. Motor cortex nodes (black) became more integrated towards the center of the network through connections with caudoputamen nodes (red). The caudoputamen played a more central role in the network, with more caudoputamen nodes functioning as hubs compared to in the control group. Network hubs consisted of 5 caudoputamen nodes (up from 3 in the control group), 9 GPe nodes (down from 12 in the control group), 3 new SNr nodes and 1 new motor cortex node. There was no network hub in the PFC in the exercise group, consistent with decreases in metabolic connectivity of the PFC with the other structures (Table 2).

**Fig. 7:**
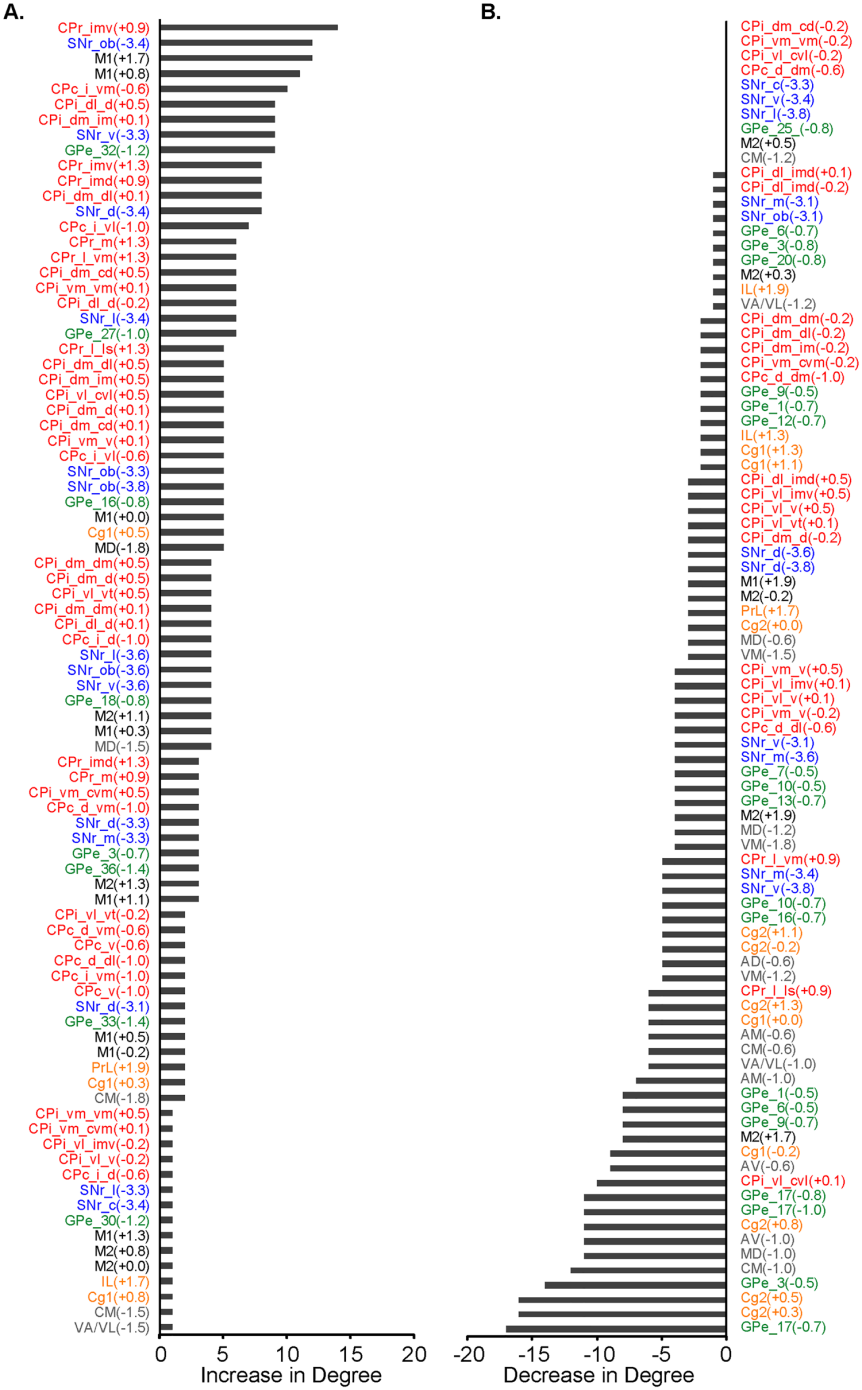
Metabolic connectivity degree changes comparing the exercise and control group. **A** Regions of interest (ROIs) showing increases in degree in the exercise compared to the non-exercise control group (*n* = 10/group). **B** ROIs showing no changes or decreases in degree in the exercise compared to control group. Bregma levels of ROIs are included in parentheses. CPr/CPi/CPc, rostral /intermediate/caudal caudoputamen, *SNr* substantia nigra pars reticulate, *GPe* globus pallidus externus, Cg1/Cg2, cingulate cortex area 1/2; *IL* infralimbic cortex, *PrL* prelimbic cortex, *M1/M2* primary/secondary motor cortex, thalamic nuclei (*AD* anterodorsal. *AM* anteromedial. *AV* anteroventral. *CM* central medial.*MD* mediodorsal. *VA/VL* ventral anterior/ventrolateral. *VM* ventromedial). For locations of ROIs see also Figs. 2, 8, 9. The number of connections linking a node to other nodes (i.e. degree) is established in the jackknife corrected network

Figure 7 shows connectivity degree changes in the exercise compared to control group of all ROIs in a ranked order. Among those with the highest gains in degree were CPr, CPi, SNr, and M1 ROIs (Fig. 7A). ROIs with the greatest losses in degree were from the Cg2, thalamus, and GPe (Fig. 7B).

**Fig. 8:**
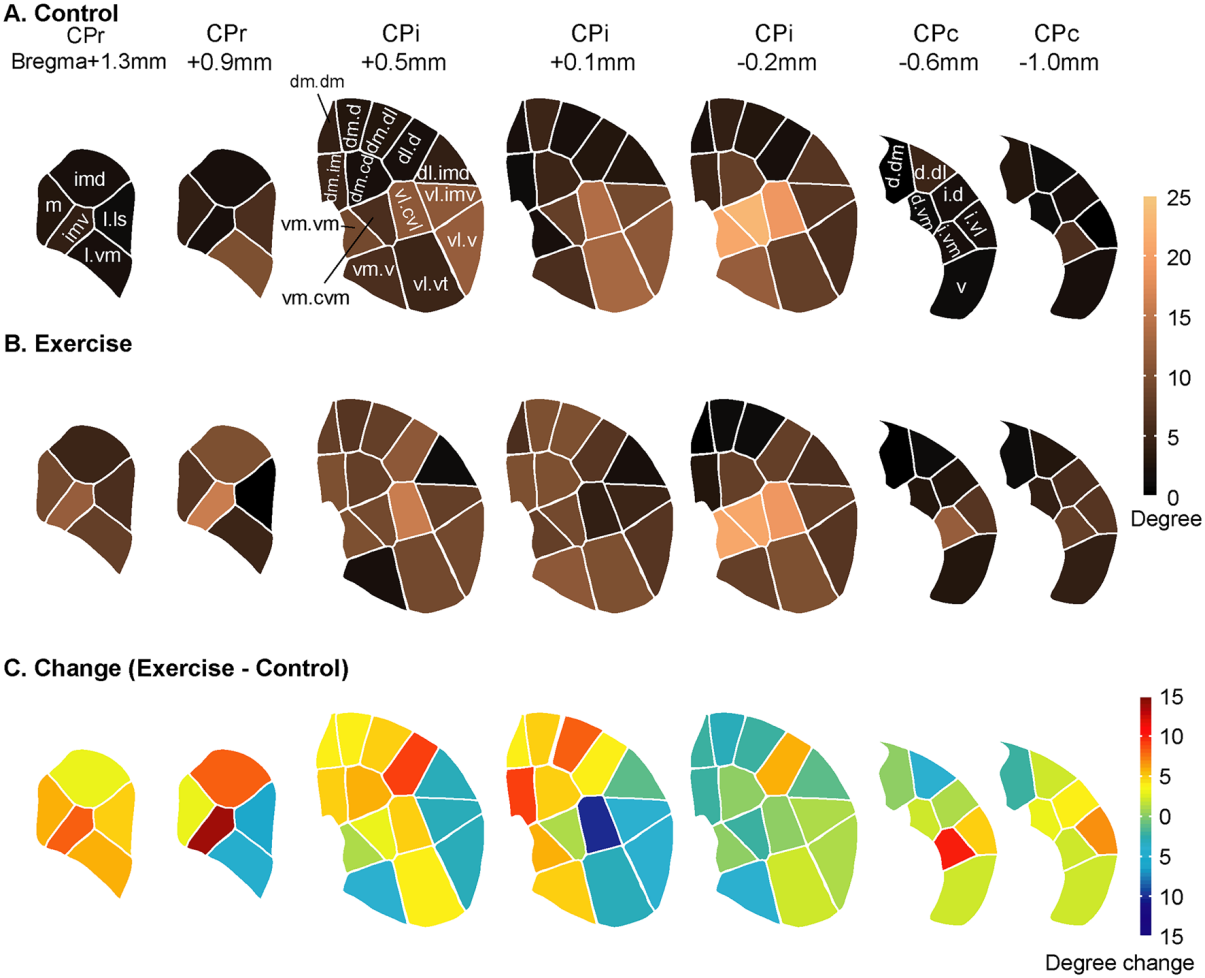
Metabolic connectivity degree changes in the caudoputamen (CP) across the cortico-basal ganglia-thalamic network. **A** Connectivity degree of CP domains in the non-exercise control group (*n* = 10) is color-coded. **B** Connectivity degree of CP domains in the exercise group (*n* = 10). **C** Connectivity degree changes in the exercise compared to control group. CPr/CPi/CPc, rostral/intermediate/caudal caudoputamen. Domain maps and nomenclature were drawn based on Hintiryan et al. (2016). The number of connections linking a node to other nodes was established in the jackknife corrected network

Figure 8 summarizes metabolic connectivity degrees of CP domains across the CBT network in the control and exercise groups, as well as exercise-associated changes along the rostral-caudal axis. The domain maps were modified from (Hintiryan et al. 2016). In the exercise compared to control group, domains showing gains in degree included most domains in the rostral CP (CPr), as well as some domains in the dorsomedial and dorsolateral areas of intermediate CP (CPi.dm, CPi.dl.d), and in the caudal CP (CPc.). Exercise-associated decreases in connectivity degree were noted in the ventrolateral areas of CP between bregma + 0.1 and + 0.9 mm, and in the medial areas at bregma − 0.2 mm.

**Fig. 9:**
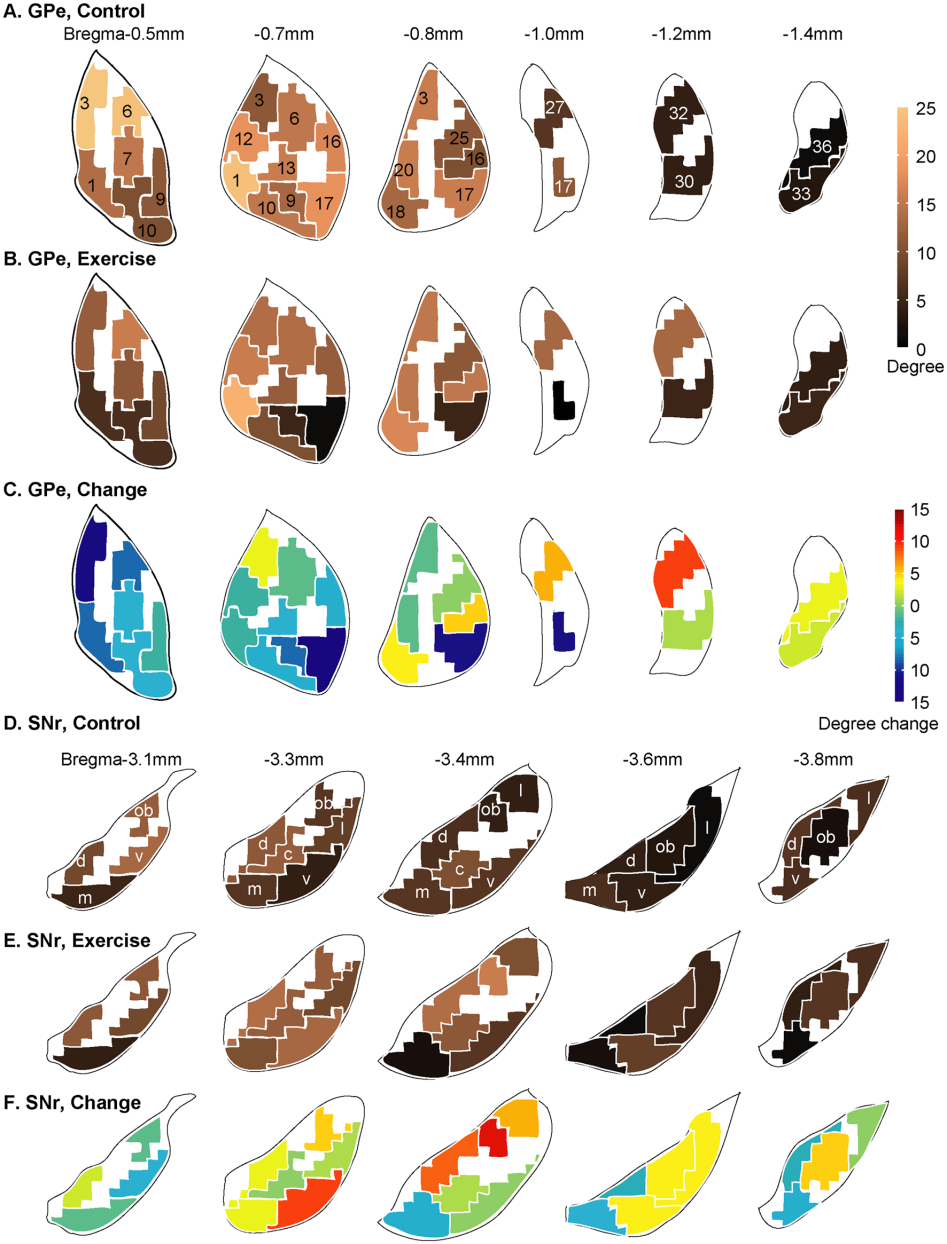
Metabolic connectivity degree changes in the globus pallidus externus (GPe) and substantial nigra pars reticulata (SNr) across the cortico-basal ganglia-thalamic network. **A** Connectivity degree of GPe domains in the non-exercise control group is color-coded (*n* = 10). **B** Connectivity degree of GPe domains in the exercise group (*n* = 10). **C** GPe connectivity degree changes comparing the exercise and control groups. **D** Connectivity degree of SNr domains in the control group. **E** Connectivity degree of SNr domains in the exercise group. **F** SNr connectivity degree changes comparing the exercise and control groups. Domain maps and domain nomenclature were drawn based on Foster et al. (2021). The number of connections linking a node to other nodes was established in the jackknife corrected network

Figure 9 summarizes metabolic connectivity degrees and exercise-associated changes in the GPe and SNr domains across the CBT network. The domain maps were modified from (Foster et al. 2021). Exercise induced overall decreases in degree in the GPe domains, except in some caudal areas (Fig. 9A–C). In the SNr (Fig. 9D–F), exercise induced largely increases in connectivity degree at intermediate and caudal levels.

## Discussion

We applied the 2DG autoradiographic cerebral metabolic mapping method to investigate exercise-associated functional reorganization in the normal mouse brain. Exercise significantly altered both regional cerebral glucose uptake in broad areas of the brain, as well as inter-regional functional interactions in the CBT network. Compared to the sedentary controls, the exercise group showed increases in positive metabolic connectivity within and between the CP and motor cortex, newly emerged negative connectivity of the SNr with GPe and CP, as well as diminished negative connectivity of the PFC with the CP. To our best knowledge, this is the first study that systematically analyzed metabolic connectivity in the CBT network at the mesoscopic level during learning of a new motor task. ROIs were chosen to conform to the subregional parcellation of the CP, SNr, and GPe in the mouse brain structural connectome. Using the structural connectome as a roadmap, the current study started to address network activity underlying the brain’s changes in learning capacity of a novel wheel walking task following chronic exercise.

### Exercise-effects on regional cerebral glucose uptake

In our study, mice underwent chronic (six weeks), high-intensity exercise on a motorized horizontal treadmill or no exercise. Cerebral metabolic mapping was thereafter undertaken in all animals during a novel wheel walking challenge, with differences in rCGU of the CBT likely reflecting long-lasting cerebral functional reorganization. Exercise resulted in broad changes in rCGU during wheel walking, including decreases in the motor areas (primary motor cortex, dorsolateral aspect of intermediate CP, SNr, zona incerta, and the cerebellar vermis); but increases broadly in the limbic areas (the hippocampus, entorhinal cortex, amygdala, hypothalamus, piriform and insular cortex, dorsal raphe, periaqueductal gray and nucleus accumbens), as well as the visual and association cortices (parietal, temporal), and dorsolateral tegmental nucleus. This general pattern of changes was remarkably similar to what we previously observed in a cerebral blood flow (CBF) mapping study (Holschneider et al. 2007). In this earlier study, rats received 6 weeks of rotarod exercise and were subsequently imaged during a locomotor challenge. Compared to sedentary controls, animals exercised on the rotarod showed decreases in regional CBF in the motor pathway (M1, M2, dorsolateral CP, zona incerta, cerebellar vermis) and primary somatosensory cortex, as well as increases in limbic regions (hippocampus, entorhinal cortex, periaqueductal gray, amygdala). Of note, the above-mentioned patterns observed after chronic exercise were generally opposite to those elicited during acute locomotion, where prior work has shown increases in rCGU (Vissing et al. 1996) and CBF (Nguyen et al. 2004) in motor regions (motor cortex, striatum, substantia nigra, cerebellum) and decreases in rCGU in limbic regions (including amygdala, hippocampus, hypothalamus, dorsal raphe). This converging evidence across species and across brain mapping modalities supports a general pattern of exercise effects on functional cerebral reorganization.

Similar effects on the motor regions have been reported after motor training in humans when comparing professional musicians and novices during the performance of finger sequences (Munte et al. 2002). The magnitude of fMRI BOLD signals to simple, overpracticed finger tasks in experts was attenuated relative to that seen in novices in the motor cortex, basal ganglia, and cerebellar vermis (Jancke et al. 2000; Kim et al. 2004; Koeneke et al. 2004). In non-musicians, attenuation of activation in somatosensory and motor cortices has been reported as subjects become more practiced on finger tasks (Morgen et al. 2004). When finger sequences are pre-learned, a lesser and more circumscribed activation has been noted in the cerebellum (vermis and hemispheres) (Friston et al. 1992; Jenkins et al. 1994), and striatum (Tracy et al. 2001). These and our findings suggest that extensive motor training results in a functionally more efficient way to control movements. The nature of this increase in functional efficacy needs further investigation, but may involve a shift from anaerobic to aerobic metabolism (McCloskey et al. 2001; Garifoli et al. 2003; Navarro et al. 2004).

### Exercise enhances metabolic connectivity of the caudoputamen and motor cortex

The notion that exercise enhanced the efficiency of the motor circuit received further support from the metabolic connectivity analysis. There was broadly increased intra- and inter-structural connectivity across the CP and motor cortex in exercised compared to control animals. The findings of increased metabolic connectivity in the CP and motor cortex in the exercised animals in the context of decreases or no change in regional CMRglu in these regions suggests that the motor regions, rather than being deactivated, were functioning with greater integration at the network level. Such dissociation between regional activity and functional coupling has been previously reported. Eisenstein et al. (2021) reported that in older adults a physically active lifestyle was associated with lower activity level of the hippocampus, but higher functional connectivity of the hippocampus to hubs of the default mode network during memory encoding. Conversely, Trujillo et al. (2015) reported that Parkinson's patients compared to healthy controls showed increased activity but decreased functional connectivity in the dorsolateral prefrontal cortex during a visuospatial task. These and other findings (Pinho et al. 2014) together suggest that a simultaneous task-related decrease in regional activity and increase in functional connectivity may be a marker for high functionality of the region, while an increase in activity coupled with a decrease in connectivity may represent a marker for dysfunction.

In the exercise compared to the control group, the rostral level and dorsal aspect of intermediate level CP showed the greatest increase in metabolic connectivity. The CPr exhibits high integration among cortical afferents, across different cortical subnetworks, suggesting cross modality integration, while CPi.dm receives input from the medial cortical subnetwork (including visual, auditory, anterior cingulate, retrosplenial, and posterior parietal association areas) (Hintiryan et al. 2016). Increased metabolic connectivity in these CP areas suggests a shift in the CP subregional recruitment that facilitates cross modality integration.

### Exercise decreases metabolic connectivity between prefrontal cortex and the basal ganglia

The prefrontal cortices project to the medial aspect of the CP and are implicated in cognitive functions, including executive function (O'Neill and Brown 2007; Baker and Ragozzino 2014; Grospe et al. 2018). While the PFC in the control group was functionally closely integrated with the basal ganglia through negative connectivity and with the motor cortex and thalamus through positive connectivity, it was functionally dissociated from these structures in the exercise group. The connectivity density of PFC with all other structures dropped in the exercise group. It is believed that the PFC is critically involved in the early phase of a motor learning task (Dayan and Cohen 2011). In fact, we have previously shown greater metabolic connectivity between the PFC and dorsomedial CP in rats during the early phase of learning a complex wheel walking task (Guo et al. 2017). As learning progresses, the PFC becomes less involved (Dayan and Cohen 2011). Wheel walking in the current study was a new motor task for the animals. Both groups were habituated to the wheel for two days prior to the 2DG mapping experiments. The finding of high connectivity to outside structures (inter-structural connectivity) in the control group is consistent with the notion that in the early phase of motor skill learning, the PFC is critically involved in cognitive control. Reduced PFC connectivity to outside structures in the exercise group, coupled with an increased metabolic connectivity of the CP and motor cortex, suggests higher network efficiency that expedites motor skill learning and the transition during initial learning from PFC to motor cortex in terms of cortical recruitment.

### Exercise led to negative metabolic connectivity between the SNr and GPe

In the basal ganglia, the CP and GPe were positively connected in both the control and exercise groups. In contrast, exercise induced substantial changes in the metabolic connectivity of SNr with CP and with GPe. In the control group, SNr-CP and SNr-GPe connectivity were relatively weak and contained both positive and negative connections. In the exercise group, SNr-CP and SNr-GPe turned exclusively negative to − 0.94% and − 3.10%, respectively. Negative connection (anticorrelation) is believed to reflect inter-regional modulation, possibly involving the suppression of excitability of a network (Gopinath et al. 2015). Though the direction of change related to excitatory-inhibitory inputs remains unresolved, negative correlations have been associated with known inhibitory connections in the rodent (Liang et al. 2012). In the indirect pathway of the basal ganglia, GPe inhibits SNr activity through inhibition of the subthalamic nucleus. The strong negative SNr-GPe connectivity, and decrease in rCGU in part of the SNr are consistent with greater activation of the indirect pathway in the exercise group.

### Translational implications

An important clinical implication of our findings is that chronic exercise may prime the brain for accelerated new motor learning, whereby CP to motor cortex networks are heavily recruited compared to CP to PFC networks. Whereas traditional motor learning theories emphasize learning specific to the context and task performed through engagement of the PFC, recent studies suggest that a general, transferable knowledge about skill learning processes that involves the CP and motor cortex, may be acquired through prior motor learning (Seidler 2004). Our prior behavioral work has demonstrated that physical exercise, in addition to improving motor function, may benefit cognitive performance (Wang et al. 2022). The extent of generalization may depend on the breadth and duration of experience obtained, the degree of arousal (Loras et al. 2020), context, and intensity (Holman and Staines 2021). Lehmann et al. (2020) showed that subjects who underwent cardiovascular exercise subsequently learned a dynamic balancing task faster compared to controls undergoing stretching. Exercise also induced increases in cerebral blood flow in frontal brain regions and changes in white matter microstructure in frontotemporal fiber tracts, suggesting a transfer potential of experience-induced brain plasticity. Inoue et al. (2018) showed that long-term exercise increased BDNF expression in the motor cortex and facilitated a transfer of motor learning from aerobic exercise to postural coordination. Aerobic exercise in stroke survivors improved cognitive domains related to motor learning (Quaney et al. 2009). It has been proposed that exercise-mediated improvements in motor learning can be mediated by discrete, experience-driven changes within specific neural representations subserving the performance of the trained task (Karni et al. 1998). However, few studies have examined the underlying functional reorganization of neural circuits. Our study highlights exercise-associated functional reorganization of the CBT circuit in areas implicated in cognitive and motor processing, which may mediate improved motor learning. As our study did not directly correlate a motor learning behavior with regional glucose uptake, such hypothesis would require further behavioral investigation. A goal for future research would be that circuit-level understanding may inform therapeutic use of exercise for the rehabilitation of patients with motor and cognitive dysfunctions.

### The importance of systematic functional connectivity analysis at the mesoscopic level

Tremendous progress has been made in understanding the structural connectome of the rodent brain (Oh et al. 2014; Zingg et al. 2014; Bota et al. 2015; Hunnicutt et al. 2016; Knox et al. 2019). Understanding of the brain functional connectome remains much less advanced (Frégnac 2017; Venkadesh and Van Horn 2021). A large part of the challenge is that brain functional connectivity is dynamic and depends on not only the current behavioral state (task, context), but also past experience (learning and memory). Advances have been made to delineate the whole-brain level functional connectome using resting-state fMRI (rs-fMRI) (Stafford et al. 2014; Mills et al. 2018; Zerbi et al. 2019; Coletta et al. 2020; Yang et al. 2021) and at the system level for a specific behavior, such as conditioned fear recall (Wheeler et al. 2013; Holschneider et al. 2014). Fregnac (2017) has emphasized that understanding mesoscale (mesoscopic) organization and full network dynamics may reveal a simpler formalism than the microscale level. Our study selected basal ganglia ROIs based on novel, mesoscopic domain definitions in the mouse brain connectome. In addition, multiple ROIs were defined at different bregma levels for some CP, GPe, and SNr domains. We felt this sampling method, informed by state-of-the-art structural connectomic data, reflects the best effort (an optimal compromise) to delineate *functional units* within brain structures. Specifically, a subregional sampling may be needed to avoid losing information when signals are spatially averaged over, for instance, whole CP, globus pallidus, or motor cortex, as many prior 2DG studies have done. At the same time mesoscopic sampling provides sufficient data simplification, while avoiding the risk of losing relevance to the interpretation of behavior through an exhaustive reductionist analysis (Frégnac 2017). In some cases, differences in metabolic connectivity were noted in the same domain across different bregma levels, e.g., CPr.l.vm (Fig. 8), suggesting the existence of multiple functional units within a domain. This may in turn inform further analysis of these domains in terms of gene expression, neurochemistry, and local circuitry.

### Limitations

Our study focused on the CBT network, with an emphasis on the basal ganglia. Brain regions outside of the sampled CBT network may also contribute to the learning and performance of the wheel walking task and undergo changes in response to exercise. These regions include the hippocampus, cerebellum, parietal association cortex, somatosensory and visual cortices, and ventral striatum (nucleus accumbens, ventral pallidum), as shown in the activation map (Fig. 3). This limitation is due to the scope and aim of the study. Mesoscopic ROI selection for the hippocampus, cerebellum, and the cortical structures remains challenging due to the large size and incomplete understanding of subregional heterogeneity of these structures, and needs to be addressed in future work.

It is important to note that correlation is not causation. Interpretation of functional connectivity between two nodes, even with direct structural connectivity, is not trivial due to the existence of indirect pathways through other node(s), possible influence from a common third node, and reciprocal connections and loops common in neural networks. Nevertheless, this mapping method provides insight into exercise-induced functional network reorganization and informs further mechanistic and causal research that focus on specific brain regions or pathways by examining neuroplasticity and manipulation.

As noted above, the functional connectome represents a dynamic map. The time scales of data sampling matter. Exploring network structure of cerebral cortex on multiple time scales, Honey et al. (2007) reported that at slower time scales (minutes), the aggregate strength of functional connectivity between regions is, on average, a good indicator of the presence of an underlying structural link. At faster time scales, significant fluctuations are observed. Thus, while the aim of anatomic parcellation remains the discovery of discrete functional units, the answers provided may depend in part on the temporal resolution of data sampling.

We did not assess the effect of age or sex in this study. These important variables need to be examined in future studies. Finally, our functional brain mapping was done during the light cycle, outside of the rodents’ typical wake cycle, which could have influenced our results. We felt such an impact was limited for several reasons. Through the course of experiment that lasted several weeks, animals were well habituated. Both treadmill and wheel running were “forced” rather than voluntary/spontaneous. And the impact was further limited by the use of the control group.

## Conclusion

Overall findings from our study support that exercise induced a significant functional reorganization of the CBT neural network that led to greater connectivity between the CP and motor cortex that may underlie gains in learning of a new motor task. Such findings support that exercise may facilitate motor learning through engagement of key motor networks important for the generalizability of motor performance and may be used to guide future rehabilitation programs.

### Supplementary Information

Below is the link to the electronic supplementary material.

Supplementary file1 (PDF 28 KB)

Supplementary file2 (TIF 617 KB)

## Data Availability

Data and codes are made available through an online database with a unique DOI under the CC-BY license. Access requests should be sent to the corresponding author and will be granted with the signing of a data sharing agreement.
