# Supplementary material for: Exercise alters cortico-basal ganglia network metabolic connectivity: a mesoscopic level analysis informed by anatomic parcellation defined in the mouse brain connectome
Source: Brain Struct Funct. 2023 Jun 12;228(8):1865–84. doi: 10.1007/s00429-023-02659-2 (PMC10516800; doi:10.1007/s00429-023-02659-2)
Supplement: Supplementary file 1 — Supplementary file1 (PDF 28 KB) [file 429_2023_2659_MOESM1_ESM.pdf]

Supplemental Fig. S1: Dot plot figure to show the data variability in rCGU between individual animals in select brain regions. Regions-of Interest (ROIs) were selected based on anatomical landmarks in the template brain. Functional ROIs were created by combining manually drawn ROIs with the significant SPM clusters ($P<0.05$ for >200 contiguous significant voxels) through logical conjunction. Mean optical density of each functional ROI was extracted for each animal using the Marsbar toolbox for SPM (version 0.42, http://marsbar.sourceforge.net/). n = 10/group.
