# Supplementary figures and images for: Exercise alters cortico-basal ganglia network metabolic connectivity: a mesoscopic level analysis informed by anatomic parcellation defined in the mouse brain connectome
Source: Brain Struct Funct. 2023 Jun 12;228(8):1865–84. doi: 10.1007/s00429-023-02659-2 (PMC10516800; doi:10.1007/s00429-023-02659-2)

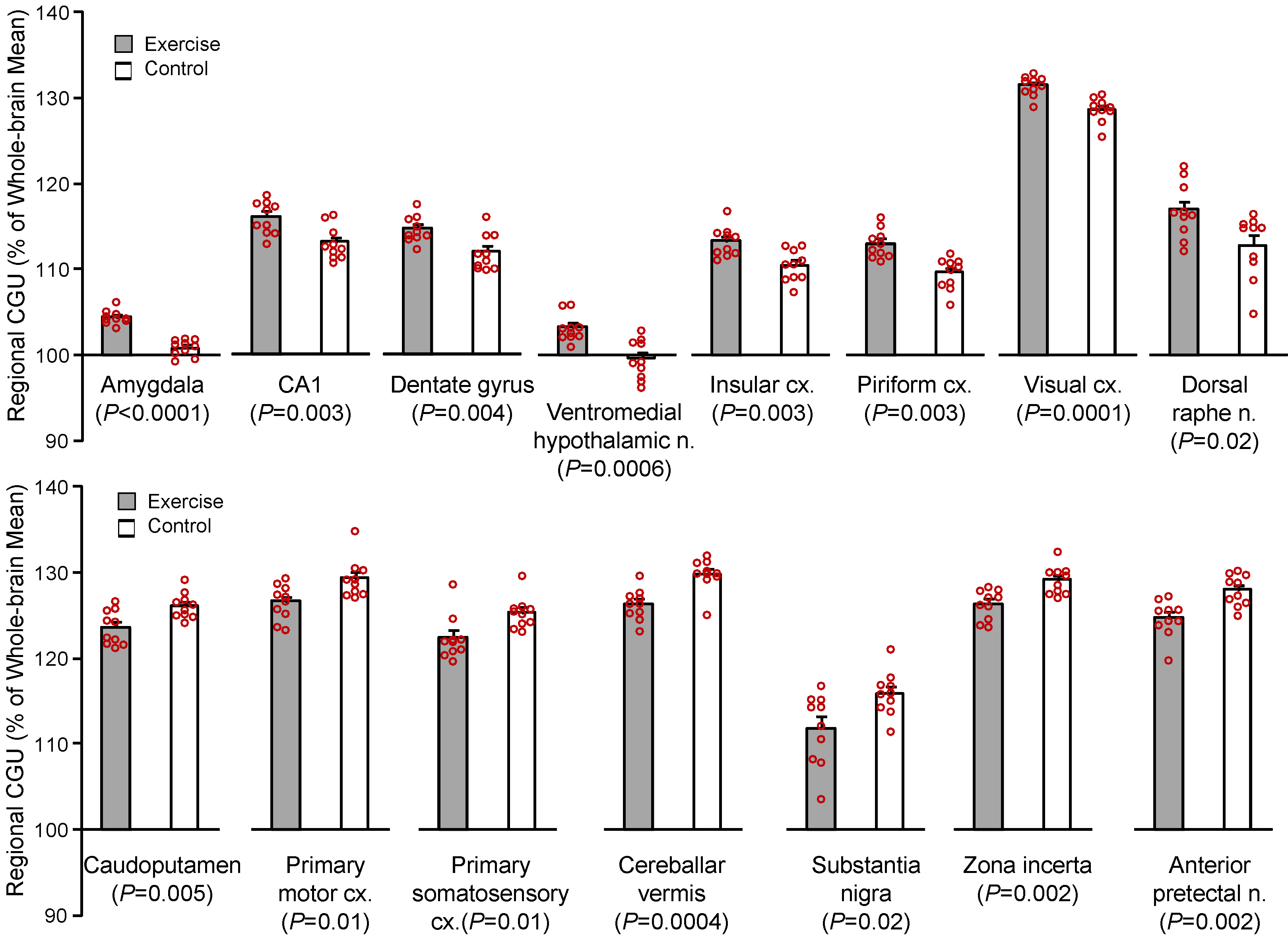

Supplement: Supplementary file 2 — Supplementary file2 (TIF 617 KB) [file 429_2023_2659_MOESM2_ESM.tif]
